# Studies on the Synthesis, Photophysical and Biological Evaluation of Some Unsymmetrical *Meso*-Tetrasubstituted Phenyl Porphyrins

**DOI:** 10.3390/molecules22111815

**Published:** 2017-10-25

**Authors:** Rica Boscencu, Gina Manda, Natalia Radulea, Radu Petre Socoteanu, Laura Cristina Ceafalan, Ionela Victoria Neagoe, Isabel Ferreira Machado, Selma Huveyda Basaga, Luís Filipe Vieira Ferreira

**Affiliations:** 1 Faculty of Pharmacy, “Carol Davila” University of Medicine and Pharmacy, 6 Traian Vuia St., 020956 Bucharest, Romania; rboscencu@yahoo.com; 2 “Victor Babeş” National Institute of Pathology, 99-101 Splaiul Independentei, 050096 Bucharest, Romania; lauraceafalan@yahoo.com (L.C.C.); neagoevictoria@gmail.com (I.V.N.); 3 “Ilie Murgulescu” Institute of Physical Chemistry, Romanian Academy, 202 Splaiul Independentei, 060021 Bucharest, Romania; 4 Centro de Química-Física Molecular, Institute of Nanosciences and Nanotechnology, Instituto Superior Técnico Av. Rovisco Pais, 1049-001 Lisboa, Portugal; ilferreiramachado@ist.utl.pt (I.F.M.); luisfilipevf@tecnico.ulisboa.pt (L.F.V.F.); 5 Polytechnic Institute of Portalegre, P-7300-110 Portalegre, Portugal; 6 Dokumar, Teknopark Blvd No 1 Pendik, 34906 Istanbul, Turkey; huveyda@sabanciuniv.edu; 7 Molecular Biology Genetics & Bioengineering Program, Faculty of Engineering & Natural Sciences, Sabanci University, Orhanli-Tuzla, 34956 Istanbul, Turkey

**Keywords:** unsymmetrical porphyrins, singlet oxygen formation quantum yield, fluorescence quantum yields, cytotoxicity, human colon carcinoma HT-29 cells, mouse L929 fibroblasts, peripheral blood mononuclear cells

## Abstract

We designed three unsymmetrical *meso*-tetrasubstituted phenyl porphyrins for further development as theranostic agents for cancer photodynamic therapy (PDT): 5-(4-hydroxy-3-methoxyphenyl)-10,15,20-*tris*-(4-acetoxy-3-methoxyphenyl)porphyrin (**P2.2**), Zn(II)-5-(4-hydroxy-3-methoxyphenyl)-10,15,20-*tris*-(4-acetoxy-3-methoxyphenyl)porphyrin (**Zn(II)2.2**) and Cu(II)-5-(4-hydroxy-3-methoxyphenyl)-10,15,20-*tris*-(4-acetoxy-3-methoxyphenyl)porphyrin (**Cu(II)2.2**). The porphyrinic compounds were synthesized and their structures were confirmed by elemental analysis, FT-IR, UV-Vis, EPR and NMR. The compounds had a good solubility in polar/nonpolar media. **P2.2** and, to a lesser extent, **Zn(II)2.2** were fluorescent, albeit with low fluoresence quantum yields. **P2.2** and **Zn(II)2.2** exhibited PDT-acceptable values of singlet oxygen generation. A “*dark*” cytotoxicity study was performed using cells that are relevant for the tumor niche (HT-29 colon carcinoma cells and L929 fibroblasts) and for blood (peripheral mononuclear cells). Cellular uptake of fluorescent compounds, cell viability/proliferation and death were evaluated. **P2.2** was highlighted as a promising theranostic agent for PDT in solid tumors considering that **P2.2** generated PDT-acceptable singlet oxygen yields, accumulated into tumor cells and less in blood cells, exhibited good fluorescence within cells for imagistic detection, and had no significant cytotoxicity in vitro against tumor and normal cells. Complexing of **P2.2** with Zn(II) or Cu(II) altered several of its PDT-relevant properties. These are consistent arguments for further developing **P2.2** in animal models of solid tumors for in vivo PDT.

## 1. Introduction

The structural diversity and wide range of biomedical applications of tetrapyrrole heterocycles have made them attractive synthetic targets over many years. The large applicability of *meso*-tetrasubstituted porphyrins and their metal complexes for therapeutical application resides in their special physicochemical and structural properties, such as intense absorption and emission in the visible region where the biological tissues absorb only weakly, high triplet state quantum yield, selectivity for tumour cells and low in vivo toxicity [1,2,3,4,5,6,7,8,9,10,11,12,13].

**Figure 1:**
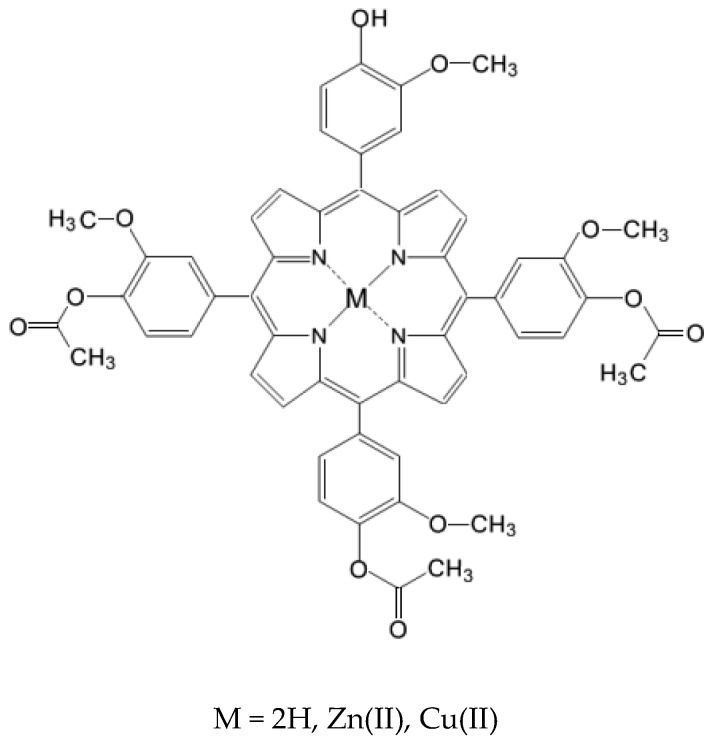
General structure of the investigated *meso*-tetrasubstituted porphyrins.

It is known that arrangement of hydrophobic and hydrophilic *meso*-substituents in tetrapyrrole structures represent an important factor which strongly influences the interaction of porphyrins with cell membranes. Therefore, changes in the molecular architecture of porphyrins by introducing polar and nonpolar substituients [14,15] or metallic ions [15] can improve their photophysical characteristics and pharmacological efficacy. Consequently, as part of our ongoing research on obtaining unsymmetrical *meso*-tetrasubstituted phenyl porphyrins [16,17,18,19,20,21,22,23,24,25,26,27,28,29], we designed and synthesized three new unsymmetrical *meso*-tetrasubstituted phenyl porphyrins whose general structure is presented in Figure 1: 5-(4-hydroxy-3-methoxyphenyl)-10,15,20-*tris*-(4-acetoxy-3-methoxyphenyl)porphyrin (**P2.2**), Zn(II)-5-(4-hydroxy-3-methoxyphenyl)-10,15,20-*tris*-(4-acetoxy-3-methoxyphenyl)porphyrin (**Zn(II)2.2**), Cu(II)-5-(4-hydroxy-3-methoxyphenyl)-10,15,20-*tris*-(4-acetoxy-3-methoxyphenyl)porphyrin (**Cu(II)2.2**). These compounds are intended to be developed as theranostic agents for photodynamic therapy (PDT) in cancer.

Our option to investigate a certain conformation of the porphyrin was imposed by the necessity of a good localization at the cellular level, which is in turn is influenced by an optimal hydrophilic/ lipophillic balance in the molecule. This particular profile could be achieved by the presence at the macrocycle periphery of several functional groups such as –OH, –OCOCH_3_, –COOH, –SO_3_H, marked by their ability to act as weak intermolecular physical bond generators that increase porphyrin solubility in polar media. One of the main difficulties was related to find the right hydrophilic/lipophilic equilibrium in the presence of different functional groups polarities, taking into account that these compounds should be soluble in biologically-friendly media and must also cross the hydrophilic cell membrane and accumulate into cells for efficient PDT. As such, the lipophillic character must not be lost, and this is the reason for selecting the compound in its A_3_B porphyrins and not A_4_ or B_4_.

The advantage of introducing the –OH group in the porphyrinic structure resides in the fact that this is a functional group with an acceptable volume that does not over-increase the molecular mass of the porphyrin. Additionally, the presence of the –OH group increased the compound solubility in PEG 200 which is a well-known and pharmacologically accepted solvent. Moreover, experimental solubility tests led us to the conclusion that our A_3_B form is more soluble in PEG than the A_4_ porphyrin type. Spectral and photophysical properties of the compounds **P2.2**, **Zn(II)2.2** and **Cu(II)2.2** were evaluated, mainly regarding singlet oxygen generation and fluorescence. Finally, a preliminary cytotoxicity study was performed using cells relevant for the tumour niche and blood, in order to select the compound that exhibits the lowest “*dark*” cytotoxicity.

## 2. Results and Discussion

### 2.1. Chemistry

The reason for selecting a specific isomer issued from synthesis was the necessity to achieve a compromise between solubility in biologically-relevant media, crossing of the lipophillic cell membrane and generation of good singlet oxygen yields for efficient PDT. These requirements were fulfilled only by the structural profile of the A_3_B isomer. In order to obtain the maximum yields of the A_3_B type mesoporphyrinic compound that exhibit a PDT-convenient hydrophilic/lipophillic balance, we chose as initial conditions a reaction mixture of 3:1 ratio for substituted benzaldehydes (4-acetoxy-3-methoxybenzaldehyde:4-hydroxy-3-methoxybenzaldehyde). Under these conditions, from the synthesis reaction resulted six porphyrin isomers (A_4_, A_3_B, A_2_B_2_-*cis* and *trans*, AB_3_ and B_4_–type), with higher percentage of the first two isomers. Their presence in the reaction product was confirmed by TLC tests.

Although the present study is mainly focused on the asymmetrical A_3_B isomer, we separated also the symmetrical A_4_ isomer in order to compare behavior of A_4_ and A_3_B at cellular level. Moreover, we selected for this study the synthesis and evaluation of A_3_B isomer complexes with zinc or copper ions. The A_4_ type metalloporphyrins have been described by us previously [19].

The unsymmetrical compounds were synthesized according the methods proposed by Adler et al. [30] and Little et al. [31] and adapted by the authors [21,24,25,26,27,28,29]. The synthesis reactions, as described at Section 3.2 and Section 3.3., were repeated several times with similar results, proving good reproducibility.

The synthesized compounds had good solubility in polar/nonpolar media (ethanol, polyethylene glycol 200, dimethylsulfoxide, dichloromethane, chloroform) and their structure were confirmed by elemental analysis, FT-IR, UV-Vis, EPR and NMR.

In the ^1^H-NMR spectra of the porphyrinic ligand **P2.2** the –NH proton signal appeared as a singlet at −2.78 ppm while in the ^1^H-NMR spectrum of **Zn(II)2.2** it could not be identified, thus confirming the coordination of the metallic ion to the nitrogen atoms of the porphyrinic core.

The ^1^H-NMR spectra showed peaks belonging to aromatic protons in the spectral range 7.10 and 7.85 ppm for **P2.2**, and between 7.05 and 7.84 for its **Zn(II)2.2** complex. Molecular asymmetry confered by the distinct type of porphyrinic substitute from *meso*-positions influenced the distribution of the NMR signals. Thus, the signal associated to protons of β-pyrrolic positions appeared at 8.96 ppm and 8.90 ppm, respectively. The O–H proton resonated at 6.23 ppm in the ^1^H-NMR, while the signal related to protons of the O–CH_3_ groups appeared as a singlet at 3.93 ppm, and that of the protons of the –OCOCH_3_ groups at 3.98 ppm.

The FT-IR spectra showed a medium strength band at ~3460 cm^−1^, which can be attributed to the functional –OH group from the structure of unsymmetrical porphyrins. The stretching frequencies at 3165 cm^−1^ confirmed the presence of N-H bonds in structure of porphyrin **P2.2**. The absence of this N-H vibration from the IR spectrum of **Zn(II)2.2** and **Cu(II)2.2** confirmed the formation of metalloporphyrins in the syntheses. Also, the FT-IR spectra of the tested compounds showed peaks at ~1587 cm^−1^ and ~1510 cm^−1^ indicating the presence of C=N and C-N bonding in the tetrapyrrolic unit. A medium absorption band in the spectral range of 1665–1692 cm^−1^ was determined by C=O stretching vibration, while bands found at ~1121 cm^−1^ can be attributed to the C–O vibration. Other bands were identified in the higher wave number region, at about 2850 cm^−1^, and are due to the stretching vibration motion of C–H bond in the –O–CH_3_ group.

The EPR spectrum of **Cu(II)2.2** was recorded on powders at room temperature. The obtained spectral values were similar to those presented in the literature for Cu(II) porphyrins with D4h coordination geometry [32,33].

### 2.2. Photophysical Characterization of Mesoporphyrinic Compounds

#### 2.2.1. UV-Vis Spectral Characterization

The UV-Vis spectral study of the proposed porphyrins was performed at room temperature, using a 2.5 × 10^−6^ M porphyrin concentration in solvents with different polarities (EtOH, PEG 200, DMSO, DCM and CHCl_3_). Data regarding spectral properties of the investigated *meso*-tetrasubstituted porphyrins are presented in Table 1.

The changes observed in the spectral behavior were in agreement with our previous results obtained for other unsymmetrical porphyrins [16,17,18,19,20,21,22,23,24,25,26,27,28,29]. Comparison of the absorption spectral parameters indicated that they were not significantly affected either by the type of peripheral substituents of the porphyrinic core, or by environmental polarity. Additionally, spectral behaviour pointed out the presence of monomeric forms at the studied concentration. The main differences appeared in the spectral behaviour of the complex vs. ligand.

The investigated porphyrins showed a higher intensity band around ~400–412 nm (Soret band), with molar absorptivity (expressed as lgε) in the range of 5.42–5.84 L·mol^−1^·cm^−1^ (Table 1). Spectral differences between the ligand **P2.2** and its metal-containing complexes consisted in the significant decrease in the Q band’s number after chelation of the porphyrinic core with metallic ions (Zn(II) or Cu(II)). Thus, **P2.2** exhibited four Q bands in the visible region 495–629 nm, while the metallated unsymmetrical porphyrins (**Zn(II)2.2** and **Cu(II)2.2**) registered two or one absorption maxima in the spectral range 527–583 nm. The Qx(0,0) max was located at ~628–629 nm, indicating that the non-metallated porphyrins still absorb significantly in the phototherapeutic window. Experimental data revealed that absorbtion maxima were less influenced by solvent polarity; for the same solvent, the absorption maxima of **Cu(II)2.2** were hypsochromically shifted, as compared to **P2.2** and **Zn(II)2.2**, due to increased conjugation occurring between the π electrons of the tetrapyrrolic unit and metallic ion electrons [34,35].

#### 2.2.2. Fluorescence Emission, Lifetime and Singlet Oxygen Formation

The current study’s aim is the development of new porphyrinic photosensitizers for PDT in cancer. Death of cancer cells is mediated by PDT-generated reactive oxygen species (ROS), particularly singlet oxygen [36].

Moreover, most porphyrinic compounds can be used also for imagistic diagnosis due to their intrinsic fluorescence properties, hence being promising theranostic candidates for solid tumors. Accordingly, fluorescence properties of the proposed new compounds were investigated, along with singlet oxygen formation quantum yields by laser photolysis experiments.

**Figure 2:**
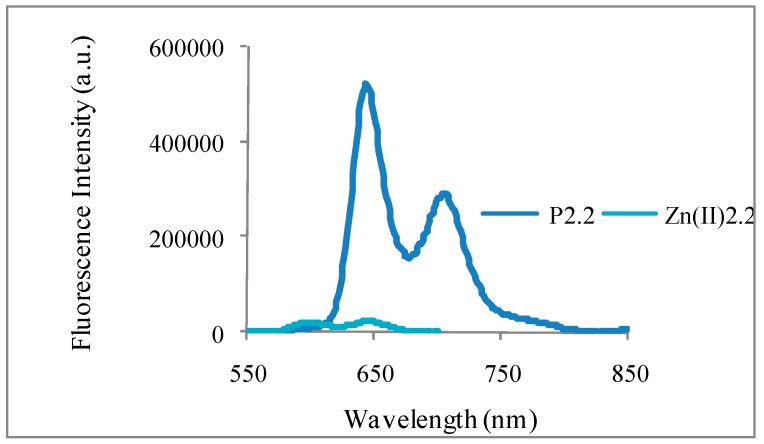
Corrected fluorescence emission spectra of **P2.2** and **Zn(II)2.2** in ethanol.

Laser-induced fluorescence emission spectra for **P2.2** and **Zn(II)2.2** dissolved in ethanol are presented in Figure 2. By comparison with TPP as reference [37,38], and using corrected emission spectra [39], results showed that **P2.2** and, to a lesser extent **Zn(II)2.2**, were fluorescent (Figure 2 and Φ_F_ in Table 1), therefore pointing to reasonable efficiency of intersystem crossing. **P2.2** and **Zn(II)2.2** were also shown to generate singlet oxygen (Φ_∆_ in Table 2). In turn, **Cu(II)2.2** exhibited no detectable fluorescence.

**Figure 3:**
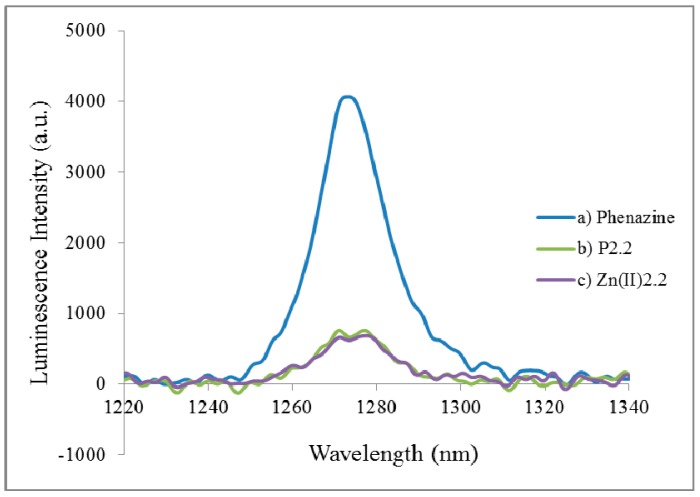
Singlet oxygem emission spectra of **P2.2** and **Zn(II)2.2** in chloroform against the reference compound (phenazine).

We evaluated the photosensitizing efficiency of the photosensitizer in chloroform, namely singlet oxygen formation quantum yields (Φ_∆_), using phenazine as standard [40]. The phosphorescence emission of singlet oxygen generated by **P2.2** and **Zn(II)2.2** at approximately 1270 nm is presented in Figure 3.

A singlet oxygen formation quantum yield of about 0.17 was evidenced for **P2.2** and **Zn(II)2.2** (Table 2), which is a reasonable Φ_∆_ value for the investigated family of compounds [37,38]. Therefore, the new mesoporphyrinic compounds proved to be promising candidates for use as photosensitizers for PDT in cancer. In turn, **Cu(II)2.2** exhibited no phosphorescence.

### 2.3. In Vitro Cytotoxicity Study

A preliminary in vitro study was performed for assessing the “*dark*” cytotoxicity profile of **P2.2**, **Zn(II)2.2** and **Cu(II)2.2** in the concentration range (0–20) μM. Considering that photosensitizers accumulate and persist in tumors, HT-29 tumor cells and L929 fibroblasts were exposed in vitro to the porphyrinic compounds for 48 h, while PBMC were treated only 24 h, presuming that the tested compounds are more quickly eliminated from blood than from tumors and tissues.

PEG 200 was used as solvent given that further nano-formulation of compounds is intended for overcoming photosensitizer aggregation in PDT. PEG is known to limit the uptake of nanostructures by blood phagocytes, hence increasing their bioavailability [41]. Various non-cytotoxic dilutions of PEG 200 were investigated (PEG, 1/4000–1/500). A distinct study was dedicated to higher PEG 200 concentrations (PEG 10× dilutions 1/400–1/50), considering that PEG may reach higher levels in blood and tissues immediately after the intravenous inoculation of the photosensitizer.

The current in vitro study takes into account that the proposed porphyrinic compounds are aimed as new and improved photosensitizers for PDT in solid tumors. Therefore, we studied in vitro their effects on cells relevant for the tumor niche [42], like the human colon carcinoma HT-29 cells and L929 mouse fibroblasts that are tumorigenic in nude mice (https://www.lgcstandards-atcc.org/products/all/CCL-1.aspx?geo_country=ro#characteristics). We have chosen colon tumour cells considering that light could be delivered directly to the target tumour through optic fibers without having to cross the skin and normal tissues to reach the tumour. We may get therefore an efficient irradiation of the tumour and minimal interaction of light with normal tissues. The in vitro action of the proposed compounds was also studied in human peripheral blood mononuclear cells (PBMC) considering that photosensitizers are generally administered by intravenous injection, and PBMC will be exposed to higher concentration of compounds.

In the “*dark*” cytotoxicity study, we analyzed the uptake of the fluorescent porphyrinic compound **P2.2** into the above mentioned cells, and the effects exerted by **P2.2** and its metal-containing complexes (**Zn(II)2.2** and **Cu(II)2.2**) on cell viability/proliferation and death.

### 2.4. In Vitro Uptake of Porphyrinic Compounds

**Figure 4:**
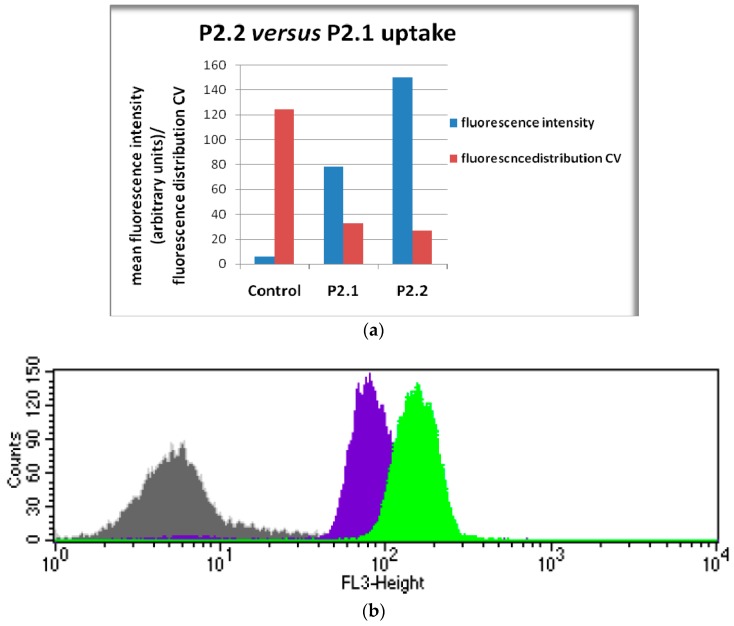
Cellular uptake of the asymmetric **P2.2** and symmetric **P2.1** compounds (10 µM) by human monocytic SC cells in 24 h culture. Cellular uptake was evaluated by flow cytometry (498 nm excitation and emmission in the red FL3 channel). Results are presented as: (**a**) mean fluorescence (geomean, arbitrary units) and the coefficient of variation (CV) of cellular fluorescence distribution; (**b**) representative histogram (**Control, P2.1, P2.2**).

The first cellular uptake study was performed by flow cytometry using the asymmetric **P2.2** compound which exhibited higher fluorescence yields than its metal-containing complexes (Table 2). The symmetric compound **P2.1**, which was also shown to be fluorescent (Table 2), was used for comparison. Experimental data (Figure 4) indicated that the intracellular fluorescence detected in human monocytic SC cells treated for 24 h with 10 μM **P2.2** was significantly higher than that of **P2.1**-treated cells in the same experimental conditions. Considering that both investigated compounds had similar fluorescence emission quantum yields (see Φ_F_ values in Table 2) we may presume that cells incorporated more the asymmetric compound **P2.2** than the symmetric **P2.1** form. This is one of the main reasons for continuing the biologic study with **P2.2**.

**Figure 5:**
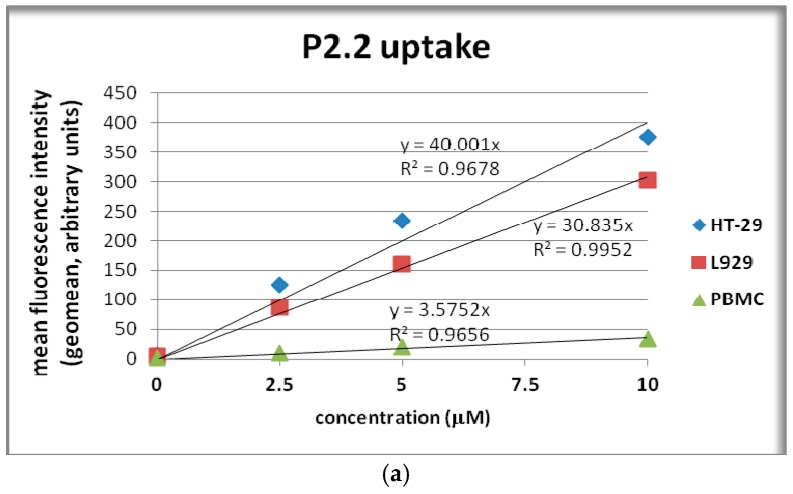

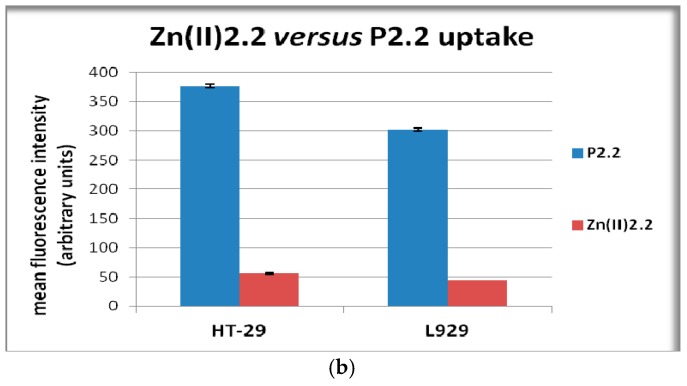
Cellular uptake of the fluorescent compound **P2.2**. (**a**) **P2.2** uptake by human HT-29 colon carcinoma cells, mouse L929 fibroblasts and human PBMC treated for 24 h with **P2.2**; (**b**) Comparison of **Zn(II)2.2** and **P2.2** uptake by HT-29 colon carcinoma cells and L929 cells treated for 24 h with porphyrinic compounds (10 μM). Cellular uptake was evaluated by flow cytometry (498 nm excitation and emmission in the red FL3 channel). Results are presented as mean ± SEM of triplicate samples.

We will further detail the uptake of the assymetric compound **P2.2**. In 24 h cultures, **P2.2** was incorporated into HT-29 colon carcinoma cells, L929 fibroblasts and PBMC in a dose-dependent manner following a linear trendline (Figure 5a). HT-29 colon carcinoma cells had the highest **P2.2** uptake, higher than L929 fibroblasts (*p* < 0.005), while PBMC exhibited the lowest uptake (Figure 5a).

The results indicated that **P2.2** could be more specifically incorporated into tumor cells, at least in HT-29 cells, and might have a higher bioavailability considering its low incorporation into PBMC. **Zn(II)2.2** was also incorporated into HT-29 carcinoma cells and L929 fibroblasts (Figure 5b), but probably due to its lower fluorescence (Figure 2), the mean fluorescence intensity of cells treated with **Zn(II)2.2** was around seven times lower than that corresponding to **P2.2**. Nevertheless, **Zn(II)2.2** could also be monitored in cells by fluorescence measurements.

**Figure 6:**
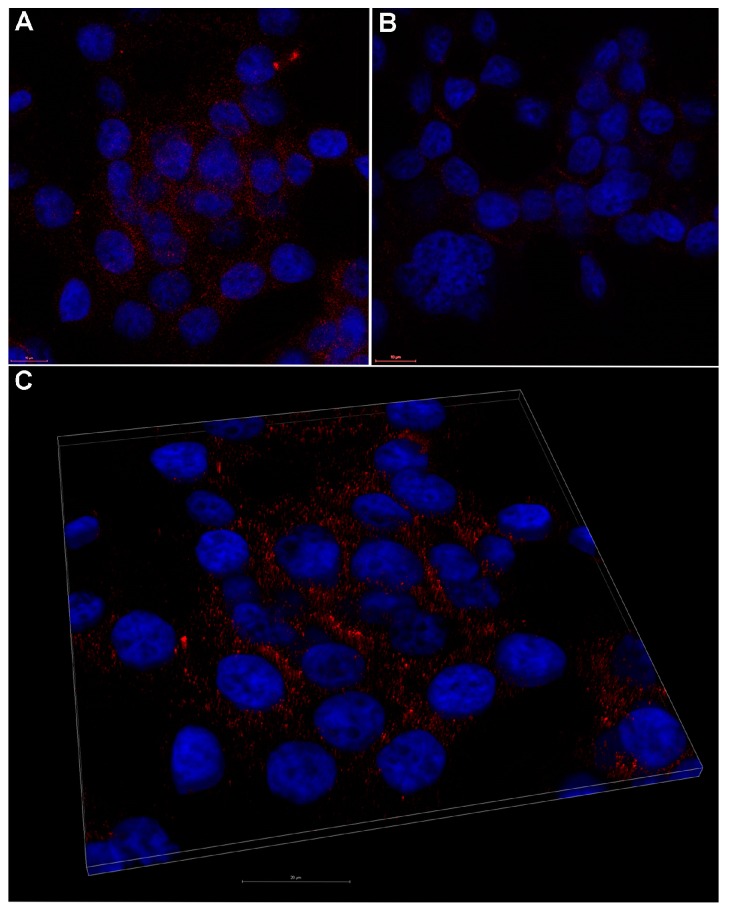
Representative laser scanning microscopy image of 10 µM **P2.2** uptake by human HT-29 colon carcinoma cells. (**A**) fluorescent **P2.2** (red) scattered throughout the cytosol as compared to control (**B**); (**C**) 3D volume rendering of a 2.36 μm z-stack from (**A**). Nuclei were stained with DAPI (blue). Scale bar 10 μm in (**A**,**B**), and 20 μm in (**C**).

A representative laser scanning microscopy image showed that 10 µM **P2.2** distributed in the cytosol of HT-29 tumor cells, most probably near the plasma membrane (Figure 6). A more detailed investigation of the subcellular localization of **P2.2** should be done in the future, as it may decisively dictate the outcome of PDT relative to the type of triggered cell death [43].

### 2.5. The In Vitro Effect of Porphyrinic Compounds on Cellular Viability and Proliferation

**Figure 7:**
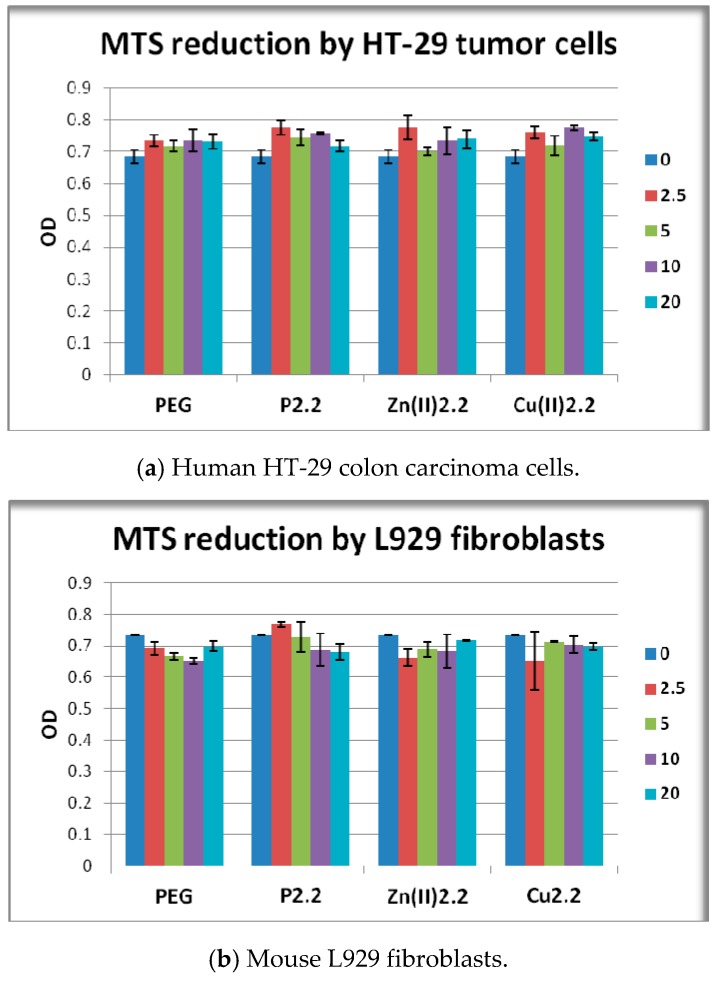

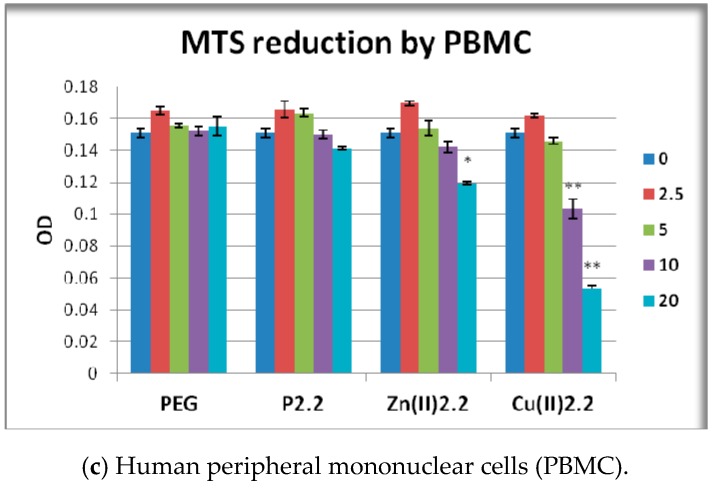
MTS reduction by HT-29 human carcinoma cells, L929 mouse fibroblasts and human PBMC exposed for 24 h (PBMC) or 48 h (HT-29 and L929 cells) to the investigated porphyrinic compounds or to solvent (PEG 200). Results are presented as mean ± SEM of triplicate samples. * *p* < 0.05, ** *p* < 0.01 as compared with PEG 200 treated cells (comparison by the Student’s *t*-test: paired two samples for mean).

Cell viability and proliferation in presence and absence of porphyrinic compounds or solvent (PEG 200) was evaluated as number of metabolically active cells using the MTS reduction test. MTS is negatively charged and does not readily penetrate cells, but it does combine with an intermediate electron acceptor (phenazine ethosulfate; PES) that can transfer electrons from the cytoplasm or plasma membrane to facilitate the reduction of tetrazolium into a colored soluble formazan product [44]. This conversion is presumably accomplished by NADPH or NADH produced by dehydrogenase enzymes in metabolically active cells [45]. By measuring the number of metabollicaly active cells, the MTS reduction test provides information on viability and proliferation of cells in culture. The proliferation capacity and viability of HT-29 tumor cells or L929 fibroblasts exposed for 48 h to porphyrinic compounds was not significantly changed as compared to solvent-treated samples (Figure 7a,b). In PBMC cultures significant differences between the effects of the tested porphyrinic compounds were registered. While **P2.2** had no major effect on the viability of PBMC, MTS reduction was significantly decreased following PBMC exposure to 20 μM **Zn(II)2.2**, or to 10–20 μM **Cu(II)2.2** (*p* < 0.05). We noted that the effect exerted by **Cu(II)2.2** on PBMC viability appeared to be more pronounced than the effect of **Zn(II)2.2**. For instance, **Cu(II)2.2** had an inhibitory effect starting at lower concentrations (10 μM) than **Zn(II)2.2** (20 μM), and the effect of 20 μM **Cu(II)2.2** was more pronounced, as observed from the decrease of MTS reduction to 68% in **Cu(II)2.2**-treated PBMC, as compared to 77% response induced by **Zn(II)2.2**.

### 2.6. The In Vitro Effect of Porphyrinic Compounds on Membrane Integrity

Membrane integrity in the presence and absence of porphyrinic compounds or solvent was evaluated by assessing the enzymatic activity of LDH in culture supernatants. LDH is a soluble yet stable enzyme found inside living cells, which is released into the extracellular space when the cell membrane is damaged. Therefore, the presence of LDH in the culture supernatant is a cell death indicator, most probably reflecting cell necrosis [46]. For accuracy, LDH release and MTS reduction were measured in the very same experimental samples.

**Figure 8:**
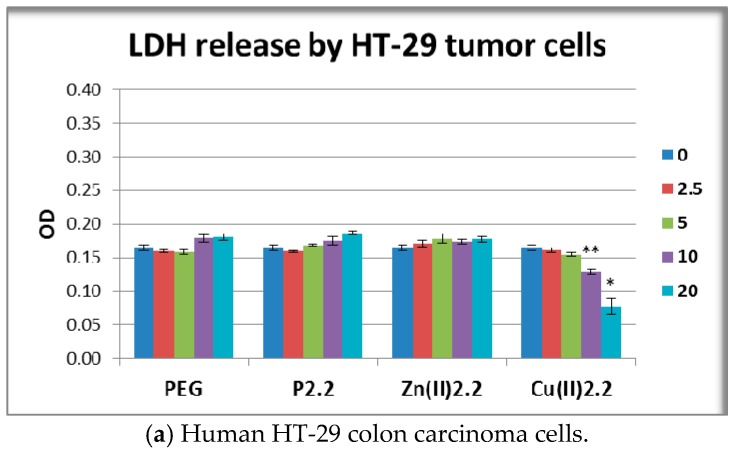

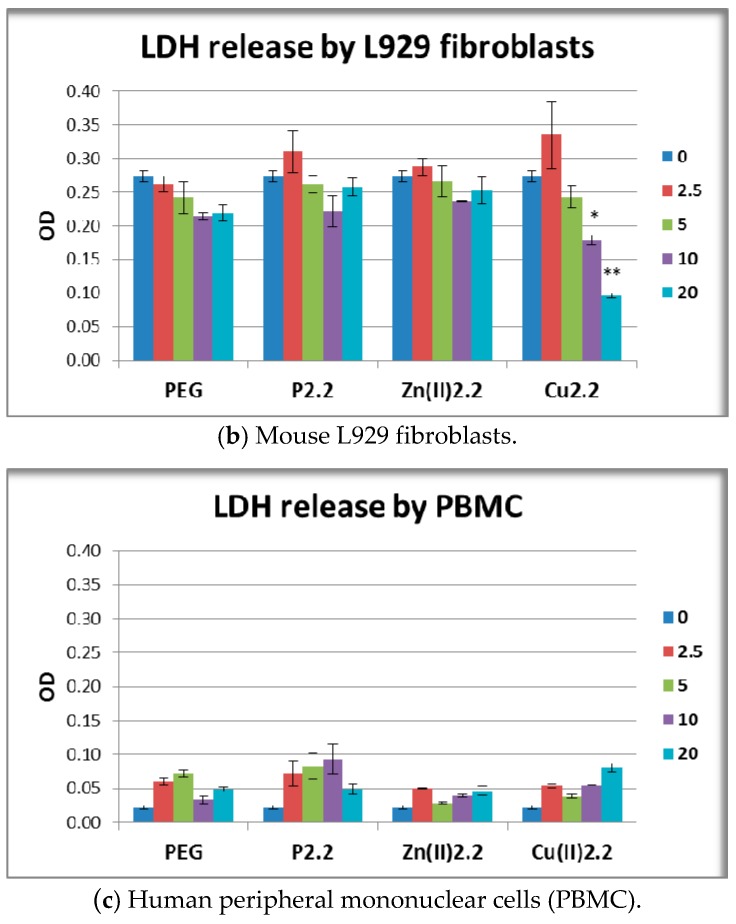
Lactate dehydrogenase (LDH) release by HT-29 human carcinoma cells, L929 mouse fibroblasts and human PBMC exposed for 24 h (PBMC) or 48 h (HT-29 and L929 cells) to the investigated porphyrinic compounds or to solvent (PEG 200). Results are presented as mean ± SEM of triplicate samples. * *p* < 0.05, ** *p* < 0.01 as compared with PEG-treated cells (Student’s *t*-test: paired two samples for mean).

**P2.2** and **Zn(II)2.2** had no significant effects on LDH release by HT-29 tumor cells and L929 fibroblasts (Figure 8a,b). These results were in good agreement with the MTS reduction data (Figure 7) which showed that **P2.2** and **Zn(II)2.2** did not alter the viability of the abovementioned cell types. Meanwhile, **Cu(II)2.2** induced an unexpected decrease of the LDH reaction in the supernatant of HT-29 and L929 cell cultures (Figure 8(a, b)), although the compound did not alter the number of metabolically active cells as measured by the MTS reduction test (Figure 7a,b). Thus, 10 μM **Cu(II)2.2** inhibited LDH release in HT-29 cell cultures to 72% of the value in solvent-treated samples (*p* < 0.01), and to 83% in L929 cell cultures (*p* < 0.05).

**Figure 9:**
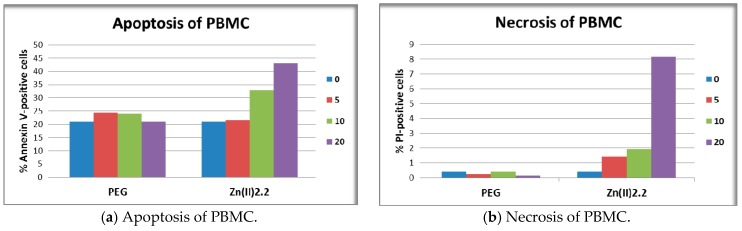
Apoptosis and necrosis of PBMC treated with **Zn(II)2.2** for 24 h, assessed by flow cytometry using the Annexin V-PI test. Annexin V-positive cells were considered apoptotic, while Annexin V-negative and PI-positive cells were considered necrotic. Fluorescence positivity was set against untreated cells.

Higher concentrations of **Cu(II)2.2** (20 μM) had an even more profound inhibitory effect, leading to the reduction of LDH response in both HT-29 tumor cells and L929 fibroblasts to around 44% from the solvent-induced effect (*p* < 0.05). This inhibitory action might be due to Cu(II)-mediated inactivation of the LDH enzyme activity [47]. In the case of PBMC, LDH release was not statistically affected by any of the investigated porhyrinic compounds (Figure 8c). It is noteworthy that the observed decrease of MTS reduction induced by 20 μM **Zn(II)2.2** (Figure 7c) was not accompanied by a significant increase of LDH release (Figure 8c). Presumably, 20 μM **Zn(II)2.2** inhibited PBMC metabolism but did not trigger significant cell death by necrosis. For obtaining more detailed information on cell death, we further investigated by flow cytometry apoptosis and necrosis of PBMC treated for 24 h with 5–20 µM **Zn(II)2.2,** using as reference cells treated with PEG 200. Experimental data (Figure 9a) indicated that **Zn(II)2.2** triggered a concentration-dependent increase of apoptotic PBMC in 24 h culture, starting with 10 µM. Necrosis was also increased (Figure 9b), but the percentage of necrotic PBMC did not exceed 8%. This is the reason why LDH release, which is less sensitive than the flow cytometry assay with propidium iodide, was not found to be significantly affected by **Zn(II)2.2** (Figure 8). Experimental results showed that 10–20 μM **Cu(II)2.2** induced a slight increase of LDH release by human PBMC (Figure 8c). Corroborating these data with the decrease of MTS reduction induced by 10–20 μM **Cu(II)2.2** (Figure 7c), we may presume that concentrations of **Cu(II)2.2** above 10 μM might be cytotoxic for human PBMC. Taking into account that **P2.2** did not alter in vitro the viability and/or proliferation of the investigated tumor and normal cells, **P2.2** emerged as a promising candidate for further development as photosensitizer for PDT in cancer.

Metal-containing porphyrinic structures (**Zn(II)2.2** and **Cu(II)2.2**) had a less convenient “*dark*” cytotoxicity profile in vitro than **P2.2**. For instance, the potential use of **Zn(II)2.2** for PDT appears to be limited by its deleterious effects exerted at higher concentration (20 μM) on the metabolism/viability of PBMC. **Cu(II)2.2** was shown to affect PBMC even more in the concentration range 10–20 μM, not only by decreasing PBMC viability/metabolism, but also by inhibiting LDH activity.

### 2.7. The In Vitro Effect of ***P2.2*** and ***Zn(II)2.2*** in Higher Concentration of PEG 200

**Figure 10:**
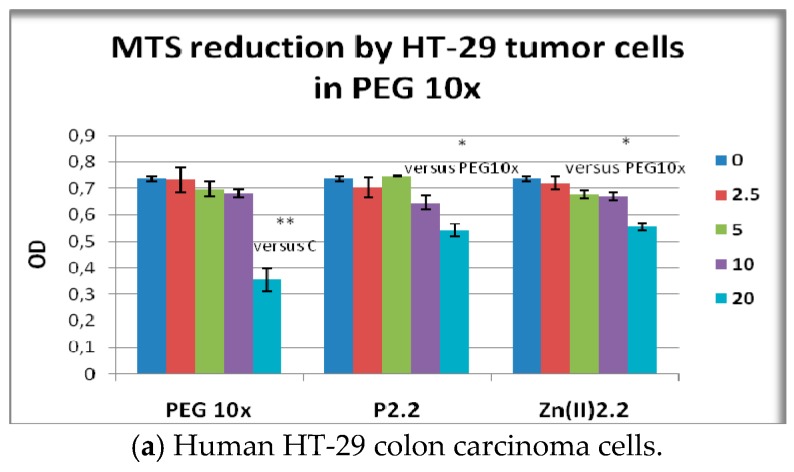

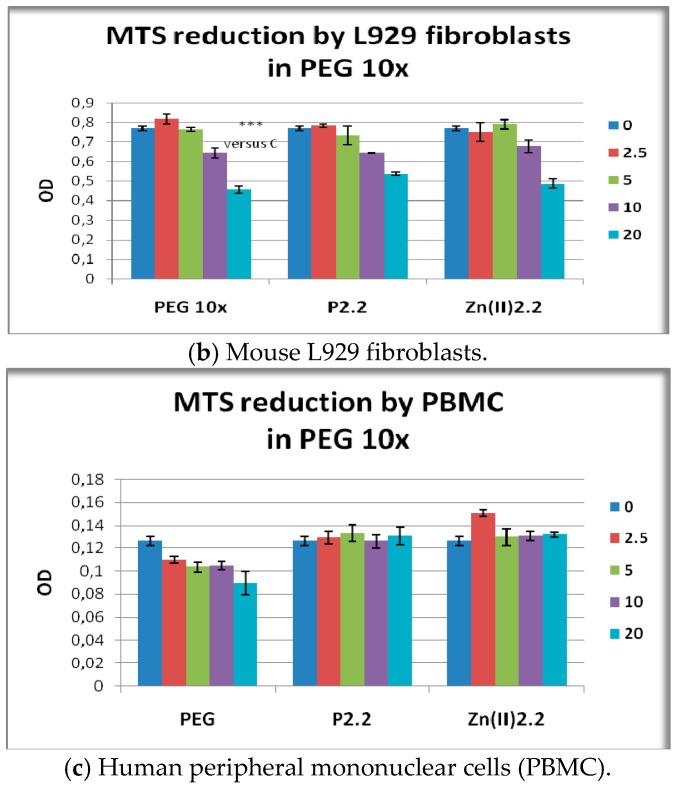
MTS reduction by HT-29 human carcinoma cells, L929 mouse fibroblasts and human PBMC exposed for 24 h (PBMC) or 48 h (HT-29 and L929 cells) to the investigated porphyrinic compounds in 10× higher PEG 200 concentrations (PEG 10×). Results presented as mean ± SEM of triplicate samples. * *p* < 0.05, ** *p* < 0.01, *** *p* < 0.001 as compared with untreated cells (C) or with PEG10x (Student’s *t*-test: paired two samples for mean).

Considering that higher concentrations of the solvent (PEG 200) may locally occur during PDT with porphyrinic compounds dissolved in PEG 200, we have investigated the effect of the previously selected compounds **P2.2** and **Zn(II)2.2** in higher PEG 200 concentration (PEG 10×, 1/400–1/50 dilution). PEG 10× (1/50) was shown to decrease the MTS reduction by HT-29 cells to 42% of the untreated cells response (*p* < 0.05) (Figure 10a), while higher PEG 200 dilutions (1/500) had no significant effects (blue PEG column in Figure 7a). A similar inhibitory effect of PEG 10× (1/50 dilution) was observed in the case of L929 fibroblasts for which MTS reduction was decreased to 59% of the untreated cells response (*p* < 0.001) (Figure 10b), and again lower PEG dilutions (1/500) had no significant effect (blue PEG column Figure 7b). Results indicated that cytotoxicity or at least inhibition of cellular metabolism may locally occur within tumors and tissues where PEG 200 may accumulate during PDT with the tested porphyrinic compounds.

PEG 10× (1/50 dilution) had only a tendency to decrease MTS reduction by PBMC, but no statistically significant difference was registered in comparison with untreated cells (Figure 10c). Accordingly, results suggest that higher PEG concentrations that could be achieved locally during intravenous inoculation of photosensitizer may not significantly alter PBMC viability/metabolism.

The effect exerted on MTS reduction by P2.2 and its Zn(II)-complex in high PEG 200 concentrations (Figure 10) generally followed the inhibitory effect of the solvent (PEG 10×). It is noteworthy that higher concentrations of P2.2 and Zn(II)2.2 (20 μM) had the tendency to protect cells against the deleterious action of PEG 10× (Figure 9a), but cellular responses generally did not reach the parameters of untreated cells.

## 3. Experimental Section

### 3.1. General Information

Commercially available chemicals and solvents were used as received from Sigma-Aldrich (St. Louis, MO, USA) and Merck (Whitehouse Station, NJ, USA). The elemental analysis of C, H and N was performed with an automatic 1108 analyzer (Carlo Erba, Milan, Italy). IR spectra were recorded with a FT-IR Tensor 27 spectrophotometer (Bruker, Fremont, CA, USA). The UV-Vis spectra of the porphyrinic compounds were recorded with a Lambda 35 spectrophotometer (Perkin-Elmer, Waltham, MA, USA). The porphyrin solutions were freshly prepared in the spectrally pure solvents (ethanol, dichloromethane, chloroform, dimethyl sulfoxide, polyethylene glycol 200) at the concentration 2.5 × 10^−6^ M and kept in dark until the measurement to prevent photodegradation. The NMR spectra were recorded with a 400 MHz Bruker NMR spectrometer and EPR spectra of the copper complex were recorded using an ART-6 Spectrometer. Fluorescence lifetimes were determined in the lifetime range from 100 ps to 3 μs using Easylife VTM equipment from OBB corporation (Birmingham, NJ, USA). This technique uses pulsed light sources from different LEDs (310 nm in this case) and measures fluorescence intensity at different time delays after the excitation pulse. In this case, 590 nm cut-off filters were used at emission both for solution and for solid samples, depending on the sample under study. The instrument response function was measured using a Ludox scattering solution. FelixGX software from OBB was used for fitting and analysis of the decay dynamics, 1 to 4 exponentials and also a lifetime distribution analysis [48], the Exponential Series Method (ESM). The schematic diagram of the LIL system is presented in reference [49]. N_2_ laser (PTI model 2000, ca. 600 ps FWHM, ~1.0 mJ per pulse), was used in laser-induced luminescence experiments. In this case the excitation wavelength was 337 nm. The light arising from the irradiation of the samples by the laser pulse was collected by a collimating beam probe coupled to an optical fiber (fused silica) and detected by an Andor ICCD, model i-Star 720 (Andor Technology Limited, Belfast, UK) gated intensified charge coupled device. The ICCD was coupled to a fixed compact imaging spectrograph (Andor, model Shamrock 163). The system can be used either by capturing all light emitted by the sample or in a time-resolved mode. The ICCD has high speed gating electronics (about 2.3 ns) and intensifier and cover at least the 250–950 nm wavelength range. Time-resolved absorption and emission spectra are available in a time range from nanoseconds to seconds. With this set-up, both fluorescence and phosphorescence spectra were easily available by the use of the variable time gate width and start delay facilities of the ICCD. The singlet oxygen measurement set-up was assembled in our laboratory. As an excitation source we use the nitrogen laser. The detector is an InGaAs CCD (model i-Dus from Andor) working at low temperature (−60 °C) coupled to a fixed spectrograph, model Shamrock 163i also from Andor [50]. Long pass filters were used to exclude totally avoid the excitation radiation from reaching the detector (LFP1000 or LFP1100 from CVI Lasers (Albuquerque, NM, USA). By comparing the total area of the emission spectra for the reference and also for the samples under study in the same solvent, with the same optical density at the excitation wavelength, the Φ_Δ_ values were obtained.

### 3.2. Synthesis of 5-(4-Hydroxy-3-methoxyphenyl)-10,15,20-tris-(4-acetoxy-3-methoxyphenyl)porphyrin 

4-hydroxy-3-methoxybenzaldehyde (1.9 g, 12.5 mmol) and 4-acetoxy-3-methoxybenzaldehyde (7.28 g, 37.5 mmol) were stirred in propionic acid (100 mL) at 100 °C. Freshly distilled pyrrole (3.45 mL, 50 mmol) was added dropwise and the mixture was stirred at 125 °C for 3 h. The crude product was cooled to room temperature, filtered off under vacuum and the precipitate was washed with water to remove traces of propionic acid. TLC test on silica gel using dichloromethane/diethyl ether (30:1 *v*/*v*) as eluent allowed identifying of isomeric structures from reaction product. The synthesis reaction provided a number of six porphyrin isomers (A_4_, A_3_B, A_2_B_2_ (*cis* and *trans*), AB_3_ and B_4_-type). In order to assess the comparative behavior at the cellular level, in this stage of the work we separate the A_4_ and A_3_B isomers. For extract and purification of the test isomers, the final product was dissolved in dichloromethane/diethyl ether (30:1, *v*/*v*), filtered and purified by column chromatography, using Al_2_O_3_ 90 (Merck, 63–200 μm 70–230 mesh) as stationary phase. The first band that passes through the chromatographic column correspond to the symmetrical porphyrin, 5,10,15,20-*meso*-tetrakis-(4-acetoxy-3-methoxyphenyl)porphyrin (**P2.1**) while the second band containing 5-(4-hydroxy-3-methoxyphenyl)-10,15,20-*tris*-(4-acetoxy-3-methoxyphenyl)porphyrin (**P2.2**). In the final stage of the synthesis proces, **P2.1** and **P2.2** porphyrins were purified by thin layer chromatography, using silica gel 60 PLC plates and dichloromethane/diethyl ether (30:1 *v*/*v*) as eluent.

*5,10,15,20-Meso-tetrakis-(4-acetoxy-3-methoxyphenyl)porphyrin* (**P2.1**): Violet crystals soluble in ethanol, dimethylsulfoxide, dichloromethane, chloroform and polyethylene glycol 200. Elemental analysis for C_56_H_46_N_4_O_12,_ calculated (found): C 69.56 (69.48), H 4.76 (4.68), N 5.79 (5.65); ^1^H-NMR, δ_H_ (CDCl_3_), ppm: −2.81 (s, 2H, -NH), 3.97 (s, 12H, O-CH_3_), 4.11 (s, 12H, OCO-CH_3_), 7,26 (s, 4H, H*_o_*_-Ph-OCOCH3_), 7,40 (d, 4H, H*_o_*_-Ph-OCOCH3_), 7,43 (d, 4H, H*_m_*_-Ph-OCOCH3_), 8,81 (d, 6H, H βpyrr); 8,92 (d, 2H, βpyrr). ^13^C-NMR, δ_C_ (CDCl_3_), ppm: 56.2, 76.7, 77.3, 110.0, 116, 119.6, 128.6, 130.0, 133.0, 134.2, 144.0, 145.2, 149.6. IR (cm^−1^): 3165, 2930, 2854, 1762, 1663, 1582, 1505, 1462, 1194, 1150, 1026, 859, 793; UV-Vis (CH_2_Cl_2_) λ (nm): 416.0, 498.4, 549.7, 586.0, 627.6.

*5-(4-Hydroxy-3-methoxyphenyl)-10,15,20-tris-(4-acetoxy-3-methoxyphenyl)porphyrin* (**P2.2**): Yield 7%; violet crystals soluble in ethanol, dimethylsulfoxide, dichloromethane, chloroform and polyethylene glycol 200. Elemental analysis for C_54_H_44_N_4_O_11,_ calculated (found): C 70.13 (70.02), H 4.76 (4.72), N 6.06 (5.94); ^1^H-NMR, δ_H_ (CDCl_3_), ppm: −2.78 (s, 2H, -NH), 3.93 (s, 3H, O-CH_3_ ), 3.98 (s, 9H, OCO-CH_3_), 4.01 (s, 9H, O-CH_3_), 6,23 (s, 1H, -OH), 7,10 (d, 3H, H*_o_*_-Ph-OCOCH3_), 7,30 (s, 1H, H*_o_*_-Ph-OCOCH3_), 7,48 (d, 3H, H*_m_*_-Ph-OCOCH3_), 7,70 (d, 1H, H*_o_*_-Ph-OH_), 7,83(s, 1H, H*_o_*_-Ph-OH_), 7,85 (d, 1H, H*_m_*_-Ph-OH_); 8,90 (d, 6H, H βpyrr); 8,96 (d, 2H, βpyrr). ^13^C-NMR, δ_c_ (CDCl_3_), ppm: 56.0, 76.7, 77.3, 116, 118.2, 119.6, 121.1, 126.0, 127.8, 128.0, 128.5, 133.0, 134.2, 146.4, 149.2, 149.6, 152.0; IR (cm^−1^): 3460, 3165, 2945, 2854, 1692, 1663, 1587, 1509, 1428, 1298, 1264, 1151, 1121, 1026, 859, 812, 732; UV-Vis (CH_2_Cl_2_) λ (nm): 402.0, 497.6, 532.8, 570.0, 626.4

### 3.3. Synthesis of M(II)-5-(4-Hydroxy-3-methoxyphenyl)-10,15,20-tris-(4-acetoxy-3-methoxyphenyl)porphyrins (***Zn(II)2.2*** and ***Cu(II)2.2***)

The synthesis of **Zn(II)2.2** and **Cu(II)2.2** was performed by refluxing a dichloromethane solution containing a mixture of 5-(4-hydroxy-3-methoxyphenyl)-10,15,20-*tris*-(4-acetoxy-3-methoxyphenyl)porphyrin (0.115 g, 0.125 mmol) and the metallic ion salts (0.023 g anhydrous zinc acetate or 0.0168 g anhydrous copper(II) chloride, respectively 0.125 mmol) in the presence of 2,6-dimethylpyridine, at 60 °C for 1 h. The presence of the porphyrinic complex in the reaction mixture was assessed by UV–Vis spectroscopy. TLC tests of the final reaction product allowed us to establish the conditions for purification of the porphyrinic complexes. The reaction products were initially purified through column chromatography (Al_2_O_3_ 90, Merck, 63–200 μm 70–230 mesh, CH_2_Cl_2_/diethyl ether 30:1 (*v*/*v*)) and finaly by TLC.

**Zn(II)2.2** was obtained with a yield of 90%, as violet crystals insoluble in water, soluble in ethanol, dimethylsulfoxide, dichloromethane, chloroform and polyethylene glycol 200. Elemental analysis for C_54_H_42_N_4_O_11_Zn: calc (found): C 65.62 (65.56), H 4.25 (4.18), N 5.67 (5.56); ^1^H-NMR, δ_H_ (CDCl_3_), ppm: 3.93 (s, 3H, O-CH_3_ ), 3.97 (s, 9H, OCO-CH_3_), 4.00 (s, 9H, O-CH_3_), 6,19 (s, 1H, -OH), 7,05 (d, 3H, H*_o_*_-Ph-OCOCH3_), 7,26 (s, 1H, H*_o_*_-Ph-OCOCH3_), 7,43 (d, 3H, H*_m_*_-Ph-OCOCH3_), 7,73 (d, 1H, H*_o_*_-Ph-OH_), 7,79(s, 1H, H*_o_*_-Ph-OH_), 7,84 (d, 1H, H*_m_*_-Ph-OH_); 8,90 (d, 6H, H βpyrr); 9,02 (d, 2H, βpyrr). 13C-NMR, δ_c_ (CDCl_3_), ppm: 56.3, 76.7, 77.4, 114.8, 117.9, 119.0, 120.0, 126.0, 127.0, 129.8, 131.5, 133.0, 134.2, 148.0, 149.2, 150.4, 152.0; IR (cm^−1^): 3462, 2923, 2852, 1665, 1645, 1581, 1510, 1468, 1334, 1274, 1162, 1108, 1024, 838, 782, 731; UV-Vis (CH_2_Cl_2_) λ(nm): 402.8, 527.6, 566.4.

**Cu(II)2.2.** was obtained with a yield of 92%, as dark red cristals insoluble in water soluble in ethanol, dimethylsulfoxide, dichloromethane, chloroform and polyethylene glycol 200. Elemental analysis for C_54_H_42_N_4_O_11_Cu: calc (found): C 65.75 (65.62), H 4.26 (4.18), N 5.68 (5.58); IR (cm^−1^): 3460, 2920, 2853, 1673, 1590, 1503, 1463, 1328, 1260, 1190, 1028, 856, 788; UV-Vis (CH_2_Cl_2_) λ (nm): 400.4, 518.4. The EPR parameters evaluated for the **Cu(II)2.2** are: g_‖_=2.233, gμ_⊥_ =2.057, A_‖_ = 158 × 10^−4^cm^−1^, α^2^ = 0.7288.

### 3.4. In Vitro “Dark” Cytotoxicity Study

For the in vitro study we used the human HT-29 colon carcinoma cell line (ATCC HTB-38™) and mouse L929 fibroblasts from subcutaneous connective tissue (ATCC CCL-1) purchased from American Type Culture Collection (ATCC, Manassas, VA, USA). Cell lines were maintained in culture in DMEM culture medium (Biochrom, Berlin, Germany) supplemented with 10% fetal bovine serum (FBS, Biochrom) and antibiotic-antimycotic solution (Sigma-Aldrich). Cell passage was performed when cells reached around 80% confluence (one-two times per week). Adherent cells were trypsinized using 0.25%/0.02%Trypsin/EDTA (Biochrom), and were replated at 0.5–1 × 10^6^ cells/25 cm^2^ flask.

Additionally, human peripheral blood mononuclear cells (PBMC) were investigated. Blood was collected from healthy volunteers in Li-heparine vacutainers. PBMC were isolated from blood by gradient density centrifugation [51], using Biocoll separation solution (Biochrom). PBMC were isolated and resuspended in RPMI 1640 medium supplemented with 10% fetal bovine serum (FBS, Biochrom) and antibiotic-antimycotic solution (Sigma-Aldrich). In some experiments we used the human monocytic SC cell line (ATCC^®^ CRL-9855™) that was grown in suspension in IMDM culture medium (Gibco, Life Technologies, Carlsbad, CA, USA) supplemented with 10% fetal bovine serum (FBS, Biochrom), antibiotic-antimycotic solution (Sigma-Aldrich), 1% HT supplement (Thermo Fisher Scientific, Waltham, MA, USA) and 0.1% 2-mercaptoethanol (Sigma). SC cells were split twice a week to keep cell density below 1 × 10^6^/mL.

Cell counting was performed by optical microscopy using a Burker-Turk counting chamber (Sigma-Aldrich, St. Louis, MO, USA). Cellular viability was assessed by the trypan blue exclusion test. Only cell suspensions with viability higher than 95% were used in experiments.

For the experiments, cell lines were plated in sterile 24 or 96 well plates, at a density of 15,000 L929 cells/cm^2^ and 30,000 HT-29 cells/cm^2^. Cells were incubated overnight at 37 °C in 5% CO_2_ for allowing their adherence. PBMC and SC cells were plated in sterile 96 well plates at a density of 1 × 10^5^ cells/well and were used immediately for experiments. *meso*-Tetrasubstituted porphyrins or solvent (PEG 200) were added to cells in solvent control samples. The final sample volume was 100 μL/well. Equivalent samples without cells were used for background assessment. All samples were incubated at 37 °C in 5% CO_2_ either for 48 h in the case of cell lines, or for 24 h in the case of PBMC.

### 3.5. Cellular Uptake of Porphyrinic Compound

For cellular uptake studies we used **P2.2**, the compound exhibiting the highest fluorescence among the three investigated mesoporphyrins. The symmetric **P2.1** compound was used for comparison. Cells were cultivated and treated with **P2.2** as described above. Adherent cells were detached with 0.25%/0.02%Trypsin/EDTA (Biochrom). Detached adherent cells or cells grown in suspension were washed twice by centrifugation with cold PBS, and were finally suspended in Live cell imaging solution (Thermo Fisher Scientific). Quantitative cellular fluorescence measurements were done within 30 min by flow cytometry (BD FACSCalibur flow cytometer, Becton Dickinson, Franklin Lakes, NJ, USA) using for excitation the 488 nm laser, while emission was recorded in the FL3 channel (red). Data were expressed as mean fluorescence intensity (geomean, arbitrary units) by processing cellular fluorescence data with the CellQuest software (BD Biosciences).

Representative images of **P2.2** uptake by cells were obtained by laser scanning microscopy. Briefly, cells were cultivated and treated with **P2.2** in on 8 chamber slides (Lab-Tek™ II Chamber Slide™ System, Nunc™, Waltham, MA, USA). At the end of cultivation, adherent cells were washed twice with warm PBS, were fixed for 15 min with FluoroFix (BioLegend, San Diego, CA, USA) and then nuclei were stained with 1.0 μg/mL 4′,6-diamidino-2-phenylindole (DAPI) (Sigma-Aldrich). Slides were mounted with Fluorescence Mounting Medium (Dako, Carpinteria, CA, USA) and were analysed on a Leica TCS SP8 Confocal laser scanning system (Leica Microsystems, Wetzlar, Germany), using an oil immersion HC PL APO CS2 63×/1.40NA objective. A 405 nm UV laser was used for imaging DAPI, whereas for **P2.2** the optimal excitation wavelength was selected using a white light laser source (which allows a tunable range between 470 and 670 nm at 1 nm intervals). Emission was registered using PMT for DAPI and transmitted light and HyD detector for **P2.2**. Image acquisition was performed using the manufacturer supplied LASX software (Leica Microsystems), and deconvolution was done with the Huygens package (Scientific Volume Imaging, Hilversum, The Netherlands).

### 3.6. Cell Viability and Proliferation

The MTS reduction test was used for assessing the number of metabolically active cells in culture (CellTiter 96^®^ AQueous One Solution Cell Proliferation Assay from Promega Corporation, (Madison, WI, USA). At the end of the treatment time, 20 μL of the kit reagent were added to each well. Cells were incubated for another 3 h at 37 °C, in 5% CO_2_ atmosphere. The optical density (OD) of samples was measured at 490 nm against the 620 nm reference, using a Sunrise ELISA reader (Tecan, Männedorf, Schweiz) complemented by Tecan’s Magellan universal reader control and data analysis software. Final OD in cellular samples was calculated by subtracting the OD of corresponding background samples (porphyrin solution in PEG 200 and culture medium, or culture medium alone). Results were presented as mean ± standard error of the mean (SEM) for OD triplicates per sample.

### 3.7. Cell Death

The LDH release test was used for assessing the rough cytotoxic effects of the tested compounds by evaluating the membrane integrity of cells using the CytoTox 96^®^ Non-Radioactive Cytotoxicity Assay (Promega Corporation). Cells were cultivated and treated with porphyrinic compounds in 96 well plates, as described above. At the end of the incubation time, culture plates were centrifuged (200 rpm, 5 min), 50 µL of supernatant were harvested and 50 µL of the kit reagent were added to supernatants. Reaction was allowed to develop for 30 min at room temperature in the dark and was then stopped using the kit stop solution. The OD of samples was measured at 490 nm using a Tecan Sunrise ELISA reader complemented by Tecan’s Magellan universal reader control and data analysis software. Final OD in cellular samples was calculated by subtracting the OD of corresponding background samples (porphyrin solution in PEG 200 and culture medium, or culture medium alone). Results were presented as mean ± standard error of the mean (SEM) for OD triplicates per sample.

Apoptosis and necrosis of PBMC was assessed by flow cytometry using the Annexin V-propidium iodide (PI) method (FITC Annexin V Apoptosis Detection Kit I, Becton Dickinson). Briefly, 10^5^ PBMC/sample were treated for 24 h with porphyrinic compounds or solvent (PEG 200), were harvested and washed twice with cold PBS and once with annexin V buffer (1200 rpm centrifugation for 5 min at 4 °C). PBMC in annexin V buffer (10^5^ cells/100 µL) were labeled with 5 µL FITC-Annexin V solution and 5 µL PI solution from the mentioned kit, at room temperature, in the dark, for 15 min. Labeling was stopped by adding 450 µL annexin V buffer. Samples were measured within 1 h using a FACSCalibur flow cytometer (Becton Dickinson). Cellular debris were eliminated from analysis by gating. At least 5000 events/sample were acquired. Data acquisition and analysis was done with the BD CellQuest software (Becton Dickinson). Annexin V-positive cells were considered apoptotic, while Annexin V-negative and PI-positive cells were considered necrotic. Positive fluorescence threshold was set against untreated cells.

## 4. Conclusions

In the current in vitro study, the newly designed porphyrinic compound **P2.2** was highlighted as a promising theranostic agent for PDT in solid tumors. **P2.2** had good solubility in biologically-friendly media, accumulated into tumor cells and less in blood cells, exhibited good fluorescence for imagistic detection, generated PDT-acceptable singlet oxygen yields, and did not exert significant cytotoxic in vitro effects on cells specific for the tumor nice (tumor colon carcinoma cells and tumorigenic fibroblasts) or blood (PBMC). These are consistent arguments for further developing **P2.2** in animal models of solid tumors, and for designing nano-formulations that may improve its efficacy and decrease unwanted side-effects. Complexing of **P2.2** with Zn(II) or Cu(II) altered its properties, as the investigated metal-containg complexes exhibited significantly lower fluorescence and ability to generate singlet oxygen in comparison to **P2.2**, and had a lower biocompatibility in relation to blood cells.

## Figures and Tables

**Table 1:** Absorption data of the mesoporphyrinic compounds in various solvents (c = 2.5 × 10^−6^ M).

Solvent	Absorption λ_max_ (nm) [lg ε] (L mol^−1^ cm^−1^)				
	Soret Band	Qy(1,0)	Qy(0,0)	Qx(1,0)	Qx(0,0)
***5-(4-hydroxy-3-methoxyphenyl)-10,15,20-tris-(4-acetoxy-3-methoxyphenyl)porphyrin***					
**CHCl_3_**	403.2[5.842]	497.0[4.152]	532.6[4.022]	570.8[3.708]	628.6[3.364]
**CH_2_Cl_2_**	402.0[5.629]	497.6[4.340]	532.8[4.073]	570.0[3.729]	626.4[3.482]
**DMSO**	404.4[5.505]	498.0[4.492]	535.2[4.330]	572.8[4.200]	629.2[3.980]
**EtOH**	400.0[5.479]	495.6[4.542]	530.8[4.400]	571.6[4.388]	627.6[4.371]
**PEG 200**	404.4[5.580]	498.0[4.630]	534.0[4.358]	572.4[3.978]	628.8[3.940]
***Zn(II)-5-(4-hydroxy-3-methoxyphenyl)-10,15,20-tris-(4-acetoxy-3-methoxyphenyl)porphyrin***					
**CHCl_3_**	407.2[5.726]	----------------	534.2[4.390]	572.5[4.270]	---------------
**CH_2_Cl_2_**	402.8[5.706]	----------------	527.6[4.459]	566.4[3.903]	---------------
**DMSO**	411.6[5.470]	----------------	542.0[4.429]	583.0[4.302]	---------------
**EtOH**	405.6[5.602]	----------------	537.6[4.450]	577.6[4.365]	---------------
**PEG 200**	409.6[5.421]	----------------	538.2[3.908]	579.5[3.602]	---------------
***Cu(II)-5-(4-hydroxy-3-methoxyphenyl)-10,15,20-tris-(4-acetoxy-3-methoxyphenyl)porphyrin***					
**CHCl_3_**	401.5[5.540]	----------------	519.6[4.634]	----------------	---------------
**CH_2_Cl_2_**	400.4[5.457]	----------------	518.4[4.343]	----------------	---------------
**DMSO**	402.4[5.436]	----------------	524.6[4.500]	----------------	---------------
**EtOH**	396.4[5.386]	----------------	516.2[4.360]	----------------	---------------
**PEG 200**	400.0[5.440]	----------------	521.2[4.455]	----------------	---------------

**Table 2:** Fluorescence emission quantum yield (Φ_F_), fluorescence lifetime (τ_F_) and singlet oxygen formation quantum yield (Φ_∆_) for the mesoporphyrins under study.

Compound	Φ_F_ *in EtOH*	τ_F_ (ns) *in EtOH*	Φ_∆_ *in CHCl_3_*
Phenazine ^(^*^)^	-	-	0.84
TPP ^(^*^)^	0.13	10.8 ± 0.1	-
P2.1	0.06	8.7 ± 0.1	0.21
P2.2	0.04	10.1 ± 0.1	0.16
Zn(II)2.2	0.002	1.7 ± 0.05	0.17

^(^*^)^ References [37,38,40].

## References

[1] Josefsen L.B., Boyle R.W. (2012). Unique diagnostic and therapeutic roles of porphyrins and phthalocyanines in photodynamic therapy, imaging and theranostics. Theranostics.

[2] Ethirajan M., Chen Y., Joshi P., Pandey R.K. (2011). The role of porphyrin chemistry in tumor imaging and photodynamic therapy. Chem. Soc. Rev..

[3] Rai P., Mallidi S., Zheng X., Rahmanzadeh R., Mir Y., Elrington S., Khurshid A., Hasan T. (2010). Development and applications of photo-triggered theranostic agents. Adv. Drug Deliv. Rev..

[4] Manivannan E., Nayan J.P., Ravindra K.P., Karl M.K., Kevin M.S., Roger G. (2011). Porphyrin-Based Multifunctional Agents for Tumor-Imaging and Photodynamic Therapy (PDT).

[5] Lovell J.F., Liu T.W.B., Chen J., Zheng G. (2010). Activatable Photosensitizers for Imaging and Therapy. Chem. Rev..

[6] Vicente M.G.H. (2001). Porphyrin-based sensitizers in the detection and treatment of cancer: Recent progress. Curr. Med. Chem..

[7] Simoes A.V.C., Adamowicz A., Dabrowski J.M., Calvete M.J.F., Abreu A.R., Stochel G., Arnaut L.G., Pereira M.M. (2012). Amphiphilic meso(sulfonate ester fluoroaryl)porphyrins: Refining the substituents of porphyrin derivatives for phototherapy and diagnostics. Tetrahedron.

[8] O’Connor A.E., Gallagher W.M., Byrne A.T. (2009). Porphyrin and nonporphyrin photosensitizers in oncology: Preclinical and clinical advances in photodynamic therapy. Photochem. Photobiol..

[9] Ortel B., Shea C.R., Calzavara-Pinton P. (2009). Molecular mechanisms of photodynamic therapy. Front. Biosci. Landmark.

[10] Yano S., Hirohara S., Obata M., Hagiya Y., Ogura S., Ikeda A., Kataoka H., Tanaka M., Joh T. (2011). Current states and future views in photodynamic therapy. J. Photochem. Photobiol. C Photochem. Rev..

[11] Musiol R., Serda M., Polanski J. (2011). Prodrugs in photodynamic anticancer therapy. Curr. Pharm. Des..

[12] Silva E.F.F., Serpa C., Dabrowski J.M., Monteiro C.J.P., Formosinho S.J., Stochel G., Urbanska K., Simoes S., Pereira M.M., Arnaut L.G. (2010). Mechanisms of singlet-oxygen and superoxide-ion generation by porphyrins and bacteriochlorins and their implications in photodynamic therapy. Chem. Eur. J..

[13] Grancho J.C.P., Pereira M.M., Miguel M.D., Gonsalves A.M.R., Burrows H.D. (2002). Synthesis, spectra and photophysics of some free base tetrafluoroalkyl and tetrafluoroaryl porphyrins with potential applications in imaging. Photochem. Photobiol..

[14] Horiuchi H., Kameya T., Hosaka M., Yoshimura K., Kyushin S., Matsumoto H., Okutsu T., Takeuchi T., Hiratsuka H. (2011). Silylation enhancement of photodynamic activity of tetraphenylporphyrin derivative. J. Photochem. Photobiol. A.

[15] Pavani C., Uchoa A.F., Oliveira C.S., Iamamoto Y., Baptista M.S. (2009). Effect of zinc insertion and hydrophobicity on the membrane interactions and PDT activity of porphyrin photosensitizers. Photochem. Photobiol. Sci..

[16] Socoteanu R., Manda G., Boscencu R., Vasiliu G., Oliveira A.S. (2015). Synthesis, Spectral Analysis and Preliminary in Vitro Evaluation of Some Tetrapyrrolic Complexes with 3d Metal Ions. Molecules.

[17] Ferreira L.F.V., Ferreira D.P., Oliveira A.S., Boscencu R., Socoteanu R., Ilie M., Constantin C., Neagu M. (2012). Synthesis, Photophysical and Cytotoxicity Evaluation of A_3_B Type Mesoporphyrinic Compounds. Dyes Pigment..

[18] Boscencu R. (2011). Unsymmetrical Mesoporphyrinic Complexes of Copper(II) and Zinc(II). Microwave-Assisted Synthesis, Spectral Characterization and Cytotoxicity Evaluation. Molecules.

[19] Boscencu R. (2012). Microwave Synthesis under Solvent-Free Conditions and Spectral Studies of Some Mesoporphyrinic Complexes. Molecules.

[20] Boscencu R., Oliveira A.S., Ferreira D.P., Ferreira L.F.V. (2012). Synthesis and spectral evaluation of some unsymmetrical mesoporphyrinic complexes. Int. J. Mol. Sci..

[21] Boscencu R., Licsandru D., Socoteanu R., Oliveira A.S., Ferreira L.F.V. (2007). Synthesis and characterization of some mesoporphyrinic compounds unsymetricaly substituted. Rev. Chim..

[22] Socoteanu R., Boscencu R., Nacea V., Oliveira A.S., Ferreira L.F.V. (2008). Microwave-assisted synthesis towards unssimetrical tetrapyrrolic compounds. Rev. Chim..

[23] Boscencu R., Socoteanu R., Ilie M., Oliveira A.S., Constantin C., Ferreira L.F.V. (2009). Synthesis, spectral and biological evaluation of some mesoporphyrinic complexes of Zn(II). Rev. Chim..

[24] Oliveira A.S., Licsandru D., Boscencu R., Socoteanu R., Nacea V., Ferreira L.F.V. (2009). A Singlet Oxygen Photogeneration and Luminescence Study of Unsymmetrically-Substituted Meso-Porphyrinic Compounds. Int. J. Photoenergy.

[25] Boscencu R., Licsandru D., Nacea V. (2008). Asymmetrically Substituted Porphyrin Derivative and Process for Obtaining the Same. National Patent.

[26] Vasiliu G., Boscencu R., Socoteanu R., Nacea V. (2014). Complex combinations of some transition metals with new unsymmetrical porphyrins. Rev. Chim..

[27] Boscencu R., Ilie M., Socoteanu R., Oliveira A.S., Constantin C., Neagu M., Manda M., Ferreira L.F.V. (2010). Microwave Synthesis, Basic Spectral and Biological Evaluation of Some Copper (II) Mesoporphyrinic Complexes. Molecules.

[28] Boscencu R., Socoteanu R., Oliveira A.S., Vieira Ferreira L.F., Nacea V., Patrinoiu G. (2008). Synthesis and characterization of some unsymmetrically-substituted mesoporphyrinic mono-hydroxyphenyl complexes of Copper(II). Pol. J. Chem..

[29] Boscencu R., Socoteanu R., Oliveira A.S., Ferreira L.F.V. (2008). Studies on Zn(II) monohydroxyphenyl mesoporphyrinic complexes. Synthesis and characterization. J. Serb. Chem. Soc..

[30] Adler A.D., Longo F.R., Finarelli J.D., Goldmacher J., Assour J., Korsakoff L. (1967). A simplified synthesis for mesotetraphenylporphine. J. Org. Chem..

[31] Little R.A., Anton J.A., Loach P.A., Ibers J.A. (1974). The synthesis of some substituted tetraarylporphyrins. J. Heter.Chem..

[32] Lin W.C., Dolphin D. (1978). Electron Spin Resonance and Electronic Structure of Metalloporphyrins. The Porphyrins.

[33] Manoharan P.T., Roger M.T., Yen T.F. (1969). ESR Study of Copper(II) and Silver(II) Tetraphenylporphyrin. Electron Spin Resonance of Metal Complexes.

[34] Gouterman M., Dolphin D. (1978). Optical Spectra and Electronic Structure of Porphyrins and Related Rings. The Porphyrins.

[35] Gouterman M., Wagniere G.H., Snyder L.C. (1963). Spectra of porphyrins: Part II. Four orbital model. J. Mol. Spectrosc..

[36] Manda G., Isvoranu G., Comanescu M.V., Manea A., Butuner B.D., Korkmaz K.S. (2015). The redox biology network in cancer pathophysiology and therapeutics. Redox Boil..

[37] Murov S.L., Carmichael I., Hug G.L. (1989). Handbook of Photochemistry.

[38] Wilkinson F., Birks J.B. (1885). Triplet quantum yields and singlet-triplet intersystem crossing. Organic Molecular Photophysics.

[39] Eaton D.F. (1989). Luminescence Spectroscopy. Handbook of Photochemistry.

[40] Rechmond R.W., Braslavsky S.E. (1988). NATO Science Series E.

[41] Karra N., Borlak J., Alonso M.J., Csaba N.S. (2012). Surface chemistry, functionalization and surface chemistry. Nanostructured Biomaterials for Overcoming Biological Barriers.

[42] Melzer C., Von Der Ohe J., Lehnert H., Ungefroren H., Hass R. (2017). Cancer stem cell niche models and contribution by mesenchymal stroma/stem cells. Mol. Cancer.

[43] Agostinis P., Berg K., Cengel K.A., Foster T.H., Girotti A.W., Gollnick S.O., Hahn S.M., Hamblin M.R., Juzeniene A., Kessel D. (2011). Photodynamic therapy of cancer: An update. CA Cancer J. Clin..

[44] Moravec R.A., Riss T.L., Niles A.L., Duellman S., Benink H.A., Tracy J., Worzella T.J., Minor L., Sittampalam G.S., Coussens N.P., Brimacombe K. (2004). Cell Viability Assays. Assay Guidance Manual [Internet].

[45] Berridge M.V., Tan A.S. (1993). Characterization of the cellular reduction of 3-(4,5-dimethylthiazol-2-yl)-2,5-diphenyltetrazolium bromide (MTT): Subcellular localization, substrate dependence, and involvement of mitochondrial electron transport in MTT reduction. Arch. Biochem. Biophys..

[46] Chan F.K.M., Moriwaki K., De Rosa M.J. (2013). Detection of Necrosis by Release of Lactate Dehydrogenase (LDH) Activity. Methods Mol. Biol..

[47] Pamp K., Bramey T., Kirsch M., De Groot H., Petrat F. (2005). NAD(H) enhances the Cu(II)-mediated inactivation of lactate dehydrogenase by increasing the accessibility of sulfhydryl groups. Free Radic Res..

[48] Branco T.J.F., Botelho do Rego A.M., Ferreira Machado I., Vieira Ferreira L.F. (2005). A luminescence lifetime distributions analysis in heterogeneous systems by the use of Excel’s Solver. J. Phys. Chem. B.

[49] Vieira Ferreira L.F., Ferreira Machado I.L. (2007). Surface photochemistry: Organic molecules within nanocavities of calixarenes. Curr. Drug Discov. Technol..

[50] Ferreira D.P., Conceição D.S., Calhelha R.C., Sousa T., Socoteanu R., Ferreira I.C.F.R., Vieira Ferreira L.F. (2016). Porphyrin dye into biopolymeric chitosan films for localizedphotodynamic therapy of Cancer. Carbohydr. Polym..

[51] Boyum A. (1968). Isolation of mononuclear cells and granulocytes from human blood. Scand. J. Clin. Lab. Investig..

